# Supplementary material for: Swine Gut Lactic Acid Bacteria and Their Exopolysaccharides Differentially Modulate Toll-like Receptor Signaling Depending on the Agave Fructans Used as a Carbon Source
Source: Animals (Basel). 2025 Apr 4;15(7):1047. doi: 10.3390/ani15071047 (PMC11988020; doi:10.3390/ani15071047)
Supplement: Supplementary file 1 [file animals-15-01047-s001.zip › animals-3490841-supplementary.pdf]

Supplementary Table S1. Features of the isolated swine LAB strains.

Bacterial strain	Individual number	Sex	Bacterial strain description	Microscopical appearance	Biochemical tests
INP_MX_001	1	Male	Long Gram-positive bacilli with diverse distribution.	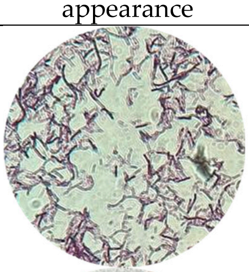	Catalase (negative) Oxidase (negative) Sporulating (negative)
INP_MX_002	1	Male	Long Gram-positive bacilli with a chain distribution.	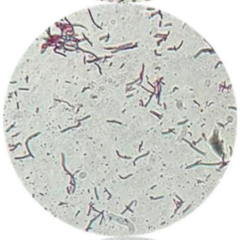	Catalase (negative) Oxidase (negative) Sporulating (negative)
INP_MX_003	2	Male	Long filamentous Gram-positive bacilli with non-uniform distribution	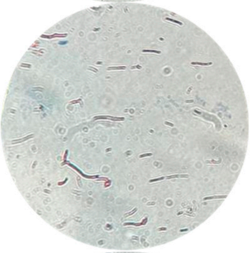	Catalase (negative) Oxidase (negative) Sporulating (negative)
INP_MX_004	3	Female	Short Gram-positive bacilli with palisade distribution.	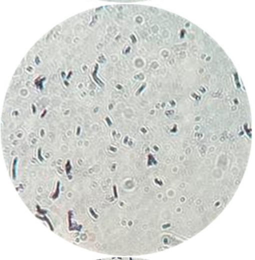	Catalase (negative) Oxidase (negative) Sporulating (negative)
INP_MX_005	4	Male	Short Gram-positive bacilli with Chinese letter distribution.	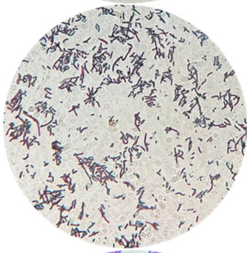	Catalase (negative) Oxidase (negative) Sporulating (negative)
INP_MX_006	5	Male	Short Gram-positive bacilli with uniform distribution.	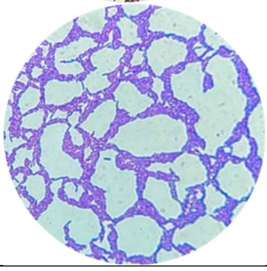	Catalase (negative) Oxidase (negative) Sporulating (negative)
